# Perceived injustice in patients with chronic pain

**DOI:** 10.1186/s13030-025-00328-w

**Published:** 2025-04-09

**Authors:** Tilman Wolter, Barbara Kleinmann

**Affiliations:** Interdisciplinary Pain Center, Medical Center, Faculty of Medicine, University of Freiburg, University of Freiburg, Breisacherstr, 117, Freiburg, 79106 Germany

**Keywords:** Chronic pain, Perceived injustice, Injustice experience questionnaire, Depression, Anxiety, Psychological factors, Social factors

## Abstract

**Background:**

Chronic pain, particularly pain secondary to trauma, is often accompanied by a feeling of perceived injustice. A prevalent feeling of injustice often goes along with a prolonged rehabilitation and problematic development of chronic pain. This feeling also correlates to catastrophizing. To date, too little is known about how the perception of injustice in chronic pain patients is interwoven with a variety of psychological, social and somatic factors. The present study sought to examine whether perceived injustice is correlated with pain level, pain diagnosis, depression, anxiety, stress, quality of life, pain related disability, occupation status and ongoing workers compensation litigation.

**Material and methods:**

During the three month period, all patients undergoing an interdisciplinary assessment of their chronic pain at our institution (*n* = 191) were asked to take part in the study. 164 patients (86%) completed the injustice experience questionnaire (IEQ). Data regarding pain level, pain diagnosis, gender, age, depression, anxiety, stress, quality of life, pain related disability, occupation status and ongoing workers compensation litigation were extracted from the patient’s charts. Correlations of these data to IEQ scores and differences between distinct subgroups of patients were analysed.

**Results:**

Median total IEQ scores were 36.0 (IQR: 29.0–42.75). Median IEQ scores for the subscale blame and severity were 14.0 (IQR: 10.0–19.5) and 21.0 (IQR: 18.0–24.0) respectively. The IEQ correlated statistically significant with anxiety, depression and stress.

No statistically significant differences were found in the IEQ scores between men and women. There was no statistically significant correlation of the IEQ scores with age, neither were statistically significant correlations with pain localizations found. No statistically significant correlation between IEQ scores and the different pain scores were found. IEQ values were higher in patients with pension application and with current sick leave. The presence of biographical factors (i.e. childhood trauma or experiences of emotional neglect) was related with higher IEQ values.

**Conclusion:**

The IEQ appears to be more closely related to psychological and social determinants of pain than to somatic factors.

## Introduction

Pain is defined as an “unpleasant sensory and emotional experience”. Chronic pain has been recognized as pain that persists past normal healing time and hence lacks the acute warning function of physiological nociception. By definition, pain is chronic if it persists for more than 3 months or recurs. Chronic pain is a frequent condition, affecting an estimated 20% of people worldwide. It is caused or maintained by a complex interplay of somatic, psychological and social factors [1].

Patients who suffer from injuries, particularly injuries with permanent physical damage, often perceive a feeling of loss. This feeling can be limiting to physical and psychological well-being and even to professional and financial independence [2, 3].This feeling of loss is often perceived as unjust, particularly when the damage is perceived as undeserved or when the injury was a consequence of a mistake or the carelessness of others [4].

Sullivan et al. [5] in 2008 first introduced the concept of perceived injustice as a contributing factor for the development of chronic pain and proposed the Injustice Experience questionnaire (IEQ) for measuring this variable. This questionnaire examines perceived injustice by investigating two contributing factors: the sense of severity/irreparability of loss and the sense of unfairness/blame. Initially, this concept was applied to pain secondary to trauma [6] and it was postulated that perceived injustice can lead to problematic pain outcomes. Since then, the IEQ has been explored in several post-traumatic pain conditions such as whiplash injury [7–9], orthopedic trauma [10] and recently also after mild traumatic brain injury [11] A recent metaanalysis found that up to 33% of all pain patients had elevated IEQ levels. Positive correlations were found to numerous psychological variables such as pain catastrophizing, posttraumatic stress, anxiety, depressive symptoms, kinesiophobia and disability while negative correlations were found to pain acceptance [12].

While the concept was initially applied to patients with posttraumatic pain**,** the IEQ meanwhile has been explored degenerative pain conditions such as hip osteoarthritis [13] or knee arthroplasty [14]. Perceived injustice has been studied also in patients with fibromyalgia [15] rheumatoid arthritis [16], musculoskeletal pain [17] and migraine [18].

Moreover, perceived injustice has been examined in populations with a variety of pain diagnoses: Carriere et al. [19] studied 344 patients following initial medical evaluation in tertiary pain center. They found a significant association between perceived injustice and opioid prescription. Among the variables pain intensity and pain behavior, depressive symptoms correlated most strongly to perceived injustice. Margiotta et al. [20] studied perceived injustice by means of the IEQ in 80 patients attending the pain clinic for the first time and having persistent pain longer than six months. They found, that one third of the patients had severely elevated IEQ scores. Patients with pain secondary to trauma were more likely to have elevated IEQ scores. La Cour et al. [21] in the same year studied 358 patients with a long lasting pain/so much a form of symptoms. The patients under study had been diagnosed either with chronic benign pain or somatoform pain disorder. Of the recorded socioeconomic data, only unemployment was found to be significantly correlated with the IEQ score, but this correlation was very weak. Strong correlations were found to the HADS (Hospital Anxiety and Depression Scale), the WHO 5 Well-Being Index and the SF 36 (Short Form Health Survey-36). Recently Alqvist Lindqvist et al. studied 65 patients referred to the University hospital with pain duration of more than three months. The study evaluated the Swedish version of the IEQ and supported structural validity and concurrent criterion validity of the Swedish version of the IEQ other measures of psychological constructs and work ability [22].

Most of the above-mentioned studies focused on the connection between perceived injustice and depression, but also anxiety, catastrophizing, disability and quality of life have also frequently been related to perceived injustice frequently. Moreover there seems to be an inverse correlation to pain acceptance [23]. Landmark et al. recently found significant associations between all psychosocial variables, including perceived unfairness, and pain-related disability in a large sample, even after adjusting for demographic factors. [24] This new appraisal of chronic pain can possibly lead to new approaches in pain treatment, although there is still little knowledge about how therapeutic interventions can reduce perceptions of in justice [6]. Therefore, it seems crucial to obtain more information on how the feeling of perceived injustice is distributed among patients with chronic pain, and with which somatic, psychological, lifestyle and social characteristics of chronic pain patients it is correlated.

To this end, we sought to add some information on whether or not and to what extent the feeling of perceived injustice is correlated to certain pain diagnoses, to functional variables such as pain- related disability and to psychological variables such as anxiety, depression and stress. We further wanted to examine particularly whether perceived injustice is related to social variables such as educational status, employment status, sick leave, gender, pension application and family status.

## Material and methods

### Patients

All patients treated in our institution with an interdisciplinary pain assessment [25] of their pain were eligible for the study. Patients with chronic pain are usually referred to our pain center by general practitioners, neurologists, orthopedists ore other medical specialists. Prior to the assessment, patients fill in the German pain questionnaire. This pain questionnaire is then evaluated by the medical staff who decides to perform an interdisciplinary assessment. During the interdisciplinary assessment, the findings from the medical and, in particular, the psychological/psychiatric interviews and the preliminary findings are compiled. Also the so called Z-codes are then classified on the basis of this assessment***.*** After the assessment the interdisciplinary team decides about the further treatment recommendation, i.e. multimodal pain treatment, interventional pain treatment or ambulatory pain treatment. The population of pain patients is characterized by a long lasting and severe pain disorder. Data were collected during a three-month period (10/1/2020–12/31/2020). Patients were asked to fill in the IEQ (injustice experience questionnaire, German version), a validated version of the IEQ in German language [26]. Patients signed a written declaration of consent to study participation.

The study was approved by the local IRB (IRB number: 20–1061). The datasets generated during and/or analyzed during the current study are available from the corresponding author on reasonable request.

### Questionnaires and chart review

The IEQ was distributed to the patients during their stay in the hospital. The questionnaire was filled in by the patients on the same day and was then returned to the staff. The IEQ consists of 12 items with a 6-point scale (1–5), so that in total maximally 60 points can be reached. Six items each form the subscale blame and the subscale severity. The cut-off values for the IEQ total score is 30; 14 for the subscale blame and 16 for the subscale severity [27]. Cronbach’s alpha for the IEQ values was α = 0.88.

General data, diagnosis based on the ICD (International Classification of Diseases), duration of pain, and medication intake were derived from the patients’ charts. Patients at our institution routinely fill in the German pain questionnaire prior to admission. From this questionnaire, which is filed in the charts, pain ratings on the 11-point numerical rating (NRS) scale and anxiety/depression/stress scores as measured by the German version [28] of the Depression, Anxiety and Stress Scale (DASS) [29] were recorded. This scale consists of 7 items each for depression, anxiety and stress. In each of these items 0–3 points can be reached. Values above 10 indicate an increased probability of the presence of an anxiety or a depressive disorder while values above 6 are suspicious for increased stress. Further, it comprises the disability score, a shortened version (3 items) of the 7-item Pain Disability Index (PDI) for the experience of impairment [30], in which scale items are rated on an 11-point scale ranging from 0–10. The mean value of these three items multiplied by 10 gives the value for the disability score. Cronbach’s alpha for the disability score was α = 0.81. Moreover, data were derives from the Marburg questionnaire on habitual health findings (FW 7), a 7 item questionnaire with a 6 point scale for each item [31]. Furthermore, data on employment status, current sick leave, pension application, highest education and marital status were collected.

A chart review included somatic and psychological diagnoses. Somatic diagnoses were further grouped according body region into the following categories: headache and facial pain, neck pain, low back pain, neuropathic pain and widespread pain.

Psychological and psychosocial diagnoses were extracted by means of the ICD-10 coding system. They were grouped into the following categories: chronic pain disorder (with somatic and psychological factors, ICD-10: F45.41) [32], depression, anxiety, sleep disorder and psychosocial factors. Psychosocial factors are coded under Z-diagnoses (factors influencing health status and contact with health services). These diagnoses were grouped in four categories: family (Z63), work (Z56), biography (Z61) and finance (Z59). For instance Z-diagnoses pertaining the family are coded in case of severe conflicts within the family. Work factors are coded, i.e. in case of imminent loss of the working place or severe conflicts at the working environment. Biographical Z-diagnoses are coded in case of childhood trauma, parental neglect or in some cases loss of parents during childhood while financial Z-diagnoses are coded in case of severe financial problems i.e. massive debts or imminent loss of housing. Moreover, medication use was extracted from the charts. A detailed analysis of the opioid medication in this sample and its conjunction to the items examined here has now been published elsewhere [33].

### Statistical analysis

A computer software package (GraphPad Prism, Version 5.01, GraphPad Software, Inc. La Jolla, USA) was used to conduct statistical analyses. Initially, descriptive statistics were applied to all measures. An unpaired t-test (in case of normally distributed variables) and in the more frequent case of missing Gaussian distribution, the Mann–Whitney Test were used the statistical significance of the differences in mean scores. A one-way ANOVA was used to calculate differences among the scores for different pain localisations. *p* < 0.05 was considered statistically significant. Pearson correlations were calculated in case of Gaussian distribution. Spearman correlations were calculated in non-normally distributed samples.

The sample size estimation was performed with G*Power [34]. Due to the different measures studied, different sample size estimations with different effect size were applied: With α = 0.05 and a power of 0.8 and an effect size of 0.3, the sample size for the one way ANOVA was estimated to be 140. For the Mann–Whitney Test with α = 0.05 and a power of 0.8 and an effect size of 0.4 the sample size was 164. Correlations with α = 0.05 and a power of 0.8 and an effect size of 0.2 had a sample size of 150.

## Results

### Patients

One hundred ninety-one patients initially fulfilled the inclusion criteria (interdisciplinary assessment between 10/1/2020 and 12/31/20200). 23 Patient denied to participate, 3 patients could not participate due to language reasons and one patient filled in the questionnaire incompletely), 164 (86%) patients were included in the analysis.

Mean age was 50.3 years and nearly two third of the patients included were female. Among the pain localizations lumbar pain (low back pain) was most frequent followed by head and face pain, cervical pain and widespread pain. The median total pain scores were 7.33 (IQR: 6.33–8.0).

The median depression score was 9.0, and the median anxiety score was 5.0 both thus lying still in the inconspicuous range. While the median stress scores were 10.0 thus being suspicious of increased stress. The median disability score was 77.33 (56.67–83.33).

Nineteen patients were retired, 31 received a disability pension, 29 patients were unemployed and 85 patients were employed. 123 patients had no ongoing pension application.

Most of the patients had a non-academic professional education, 27 patients (16%) had an academic education while 10 patients (6%) had no professional education at all.

One hundred patients were married, 13 patients were divorced, 48 patients were unwedded and 3 patients were widowed. (Table 1).

**Table 1:** Patient characteristics

		Patients (n)
Age^a^		50.3 (SD 14.2)
Sex (m/f)		67/97
Pain localization	Head and Face	30 (18.3%)
	Cervical	23 (14.0%)
	Lumbar	66 (40.3%)
	Extremities	15 (9.1%)
	Abominal	5 (3.0%)
	WSP	25 (15.2%)
Occupational Status	Retired:	19 (11.5%)
	Disability pension:	31(18.9%)
	Unemployed:	29 (17.6%)
	Employed:	85 (51.9%)
Work leave	Yes:	58 (35.7%)
	No:	66 (40.2%)
	n.a.:	40 (24.4%)
Pension application	Yes:	12 (7.3%)
	No:	123 (75.0%)
	n.a.:	29 (17.6%)
Professional education	Academic:	27 (16.4%)
	Non-academic:	128 (78.0%)
	None:	10 (6%)
Marital status	married:	100 (60.9%)
	divorced:	13 (7.9%)
	widowed:	3 (1.8%)
	unwedded:	48 (29.3%)
Coded psychological diagnoses:	Patients (n)	Patients (n)
F45.41	Yes: 149	No: 15
Depression	Yes: 79,	No: 85
Anxiety	Yes: 12	No: 152
Somatization disorder	Yes: 7	No: 157
Sleep disorder	Yes: 83	No: 81
Coded Z-diagnoses		
Family	Yes: 52	No: 112
Work	Yes: 88	No. 76
	Yes: 37	No: 127
Finance	Yes: 26	No. 138
Any Z-diagnose	Yes: 129	No: 35
Pain scores		Median (IQR)
	Current	7.0 (5.0–8.0)
	Mean^a^	7.0 (6.0–8.0)
	Highest	9.0 (8.0–10.0)
	Bearable	3.0 (2.0–4.0)
	Total^b^	7.33 (6.33–8.0)
IEQ	Blame	8.0 (4.0–13.75)
	Severity	15.0 (12.0–18.0)
	Total	24.0 (17.0–31.0)
DASS	Depression	9.0 (4.0–14.0)
	Anxiety	5.0 (2.0–9.0)
	Stress	10.0 (7.0–14.0)
	Total	25.0 (15.0–34.0)
FW 7		10.0 (4.0–14.75)
Disability score		77.33 (56.67–83.33)

Personal data, pain localizations, socioeconomic data, coded diagnoses and scales: *WSP *wide spread pain, *IEQ *Injustice experience questionnaire, *DASS *Depression, Anxiety and Stress Scale, F45.41 = ICD-10 code for pain disorder with somatic and psychological factors, FW7 = Marburg questionnaire on habitual health findings

^a^during the last 4 weeks

^b^total = (current + mean + highest)/3

### Perceived injustice

Median total IEQ scores were 24.0 (IQR: 17.0–31.0. Median IEQ scores for the subscale blame and severity were 8.0 (IQR: 4.0–13.75) and 15.0 (IQR: 12.0–18.0) respectively. 122 patients (74.3%) scored 30.0 or below in the IEQ total, while 42 patients (25.6%) scored above. 34 patients (20.7%) scored above 14 for the IEQ subscale blame while 69 (42.1%) scored above 16 for the IEQ subscale severity.

### Perceived injustice and somatic factors

No statistically significant differences were found in the IEQ scores between men and women. There was no statistically significant correlation of the IEQ scores with age (Table 2).

**Table 2:** Correlation of IEQ scores with age (years) r = spearman r, comparison of IEQ values, median and interquartile ranges in parenthesis, in male (m) and female (f) patients, * Mann Whitney test,, *p* < 0.05 = significant

		IEQ total	IEQ blame	IEQ severity
Age	50.3 (SD 14.2)	r = 0.01561, *p* = 0.842	r = 0.06506, *p* = 0.408	r = −0.04126, *p* = 0.600
		IEQ total	IEQ blame	IEQ severity
		median (IQR)	median (IQR)	median (IQR)
Sex (m/f)*	67/97	m: 24.0 (17.0–31.0) f: 23.0 /(16.0- 31.5) *p* = 0.901	m: 9.0 (4.0–14.0) f: 8.0 (4.0- 13.5) *p* = 0.967	m: 16.0 (12.0–18.0) f: 15.0 (12.0- 19.0) *p* = 0.971

Nor were statistically significant correlations with pain localizations found (Table 3). Patients with headaches mostly suffered from migraine or chronic tension headaches, while patients with lumbar and cervical pain mostly suffered from degenerative spine disease. Patients with abdominal pain often suffered from pelvic floor pain, but also patients with abdominal adhesions. Patients with WSP mostly suffered from fibromyalgia syndrome. Patients with pain in the extremities had CRPS in the majority of cases, and in some cases also neuropathic pain.

**Table 3:** 1-Way ANOVA (Kruskal–Wallis test) of the IEQ total and subscales (in patients with different pain localizations: median and interquartile ranges (IQR) in parenthesis

Pain localization	IEQ total	IEQ blame	IEQ severity
	median (IQR)	median (IQR)	median (IQR)
Head and Face	23.0 (14.25–30.25)	8.0 (2.75–13.25)	14.0 (8.75–18.0)
Cervical	25.0 (17.0–31.0)	9.0 (6.0–12.0)	15.0 (11.0–19.0)
Lumbar	24.0 (17.75–30.75)	9.0 (4.0–13.25)	15.0 (12.0–17.25)
Extremities	27.0 (22.0–33.0)	10.0 (4.0–15.0)	17.0 (16.0–19.0)
Abdominal	19.0 (12.5–31.5)	6.0 (2.5–14.0)	12.0 (10.0–18.0)
WSP	19.0 (15.0–33.0)	7.0 (3.5–14.5)	12.0 (10.0–19.0)
	*p* = 0.562	*p* = 0.956	*p* = 0.166

*WSP *wide spread pain

No statistically significant correlation between IEQ scores and the different pain scores were found. The IEQ correlated in a statistically significant manner with pain disability (Table 4).

**Table 4:** Correlations between IEQ and pain scores

Pain scores	IEQ total	IEQ blame	IEQ severity
Current	r = 0.07711, *p* = 0.330	r = 0.08371, *p* = 0.288	r = 0.04137, *p* = 0.600
Mean^a^	r = 0.1435, *p* = 0.068	r = 0.1787, *p* = 0.023	r = 0.09479, *p* = 0.230
Highest	r = 0.02528, *p* = 0.749	r = 0.04826, *p* = 0.542	r = 0.01525, *p* = 0.8477
Bearable	r = 0.01518, *p* = 0.850	r = 0.05251, *p* = 0.512	r = −0.01736, *p* = 0.829
Total NRS	r = 0.1141, *p* = 0.146	r = 0.1246, *p* = 0.112	r = 0.08915, *p* = 0.256

^a^during the last 4 weeks, r = spearman r, *p* < 0.05 = significant

### Perceived injustice and psychological factors

The IEQ had a statistically significant positive relationship with anxiety, depression and stress. Also the DASS total score correlated with the IEQ (Table 4). The coded diagnoses of depression and pain disorder with somatic and psychological factors were associated with elevated IEQ values (Table 5).

**Table 5:** Correlations of the IEQ with psychological factors as measured by the DASS, the PDI and the FW7, comparisons of median values (interquartile ranges in brackets) of patients with and without coded psychological diagnoses, F45.41 = pain disorder with somatic and psychological factors (Mann–Whitney test)

	IEQ total	IEQ blame	IEQ severity
DASS			
Depression	r = 0.6125, *p* < 0.001	r = 0.5773, *p* < 0.001	r = 0.5576, *p* < 0.001
Anxiety	r = 0.5033, *p* < 0.001	r = 0.4864, *p* < 0.001	r = 0.4426, *p* < 0.001
Stress	r = 0.4981, *p* < 0.001	r = 0.4895, *p* < 0.001	r = 0.4313, *p* < 0.001
Total	r = 0.6189, *p* < 0.001	r = 0.5943, *p* < 0.001	r = 0.5499, *p* < 0.001
PDI	r = 0.1132, *p* = 0.149	r = 0.1004, *p* = 0.201	r = 0.1165, *p* = 0.137
FW 7	r = −0.2927, *p* < 0.0001	r = −0.2741, *p* = 0.0014	r = −0.2831, *p* < 0.001
Coded diagnoses			
F45.41	Yes 25.0 (17.0–32.0) No 17.0 (14.0–20.0) , *p* < 0.001	Yes 9.0 (4.0–14.0) No 5.0 (4.0–7.0) *p* = 0.011	Yes 16.0 (12.0–18.0) No 11.0 (8.0–12.0) *p* = 0.001
Depression	Yes 28.0 (21.5–34.0) No 20.0 (14.0–27.0) *p* < 0.001	Yes 12.0 (7.5–15.0) No 6.0 (3.0–11.0) *p* < 0.001	Yes 17.0 (13.0–20.0) No 14.0 (10.0–17.0) , *p* < 0.001
Anxiety	Yes 29.0 (20.0–35.0) No 24.0 (17.0–30.0) *p* = 0.190	Yes 12.0 (5.5–17.5) No 8.0 (4.0–13.0) *p* = 0.186	Yes 16.0 (12.0–19.5) No 15.0 (12.0–18.0) *p* = 0.342
Sleep disorder	Yes 24.0 (17.0–30.0) No 22.0 (16.0–31.0) *p* = 0.448	Yes 9.0 (5.0–13.0) No 8.0 (4.0–14.0) *p* = 0.640	Yes 16.0 (12.0–128.0) No 14.0 (11.0–18.0) *p* = 0.303

### Perceived injustice and social factors

The IEQ values were higher in patients with pension application and with current sick leave than in those patients without. The difference was statistically significant. The difference in IEQ scores in different educational levels was statistically significant in the IEQ total and in the IEQ severity subscale, but not in the IEQ blame subscale. Among the coded psychosocial diagnoses, statistically significant differences of the IEQ total and the subscales blame and severity were only found between patients with and without the presence biographical factors (Table 6).

**Table 6:** IEQ and psychosocial factors, dichotomous variables: Mann Whitney test, other variables: Kruskal Wallis test

	IEQ total	IEQ blame	IEQ severity
Occupational status			
Employed	21.0 (15.0–29.0)	7.0 (3.0–12.0)	15.0 (10.0–18.0)
Unemployed	25.0 (19.0–36.25)	9.5 (5.75–14.5)	15.0 (12.0–19.25)
Retired	25.5 (16.75–30.0)	9.0 (5.75–15.0)	16.0 (11.75–18.0)
Disability pension	27.0 (18.25–33.5)	10.5 (3.75–15.25	16.0 (13.75–19.25)
	*p* = 0.151	*p* = 0.185	*p* = 0.291
Work leave	Yes 24.5 (17.0–33.00) No 20.0 (14.0–27.0) *p* = 0.010	Yes 9.5 (4.0–15.0) No 6.0 (2.75–12.0) *p* = 0.006	Yes 16.0 (11.0–19.0) No 13.0 (10.0–17.0) *p* = 0.0438
Pension application	Yes 28.0 (24.0–38.75) No 29.0 (17.0–29.0) *p* = 0.022	Yes 11.5 (8.25–16.75) No 8.00(4.0–13.0) *p* = 0.024	Yes 18.0 (14.25–28.0) No 15.0 (12.0–1.0) *p* = 0.031
Professional education			
Academic	17.0 (12.0–26.0)	6.0 (3.0–11.0)	12.0 (9.0–16.0)
Non academic	24.5 (17.25–32.0)	9.0 (4.25–14.0)	16.0 (12.0–18.0)
None	19.0 (16.5–24.5)	7.0 (4.0–12.0)	13.0 (11.0–14.0)
	*p* = 0.014	*p* = 0.096	*p* = 0.002
Marital status			
Divorced	26.0 (14.0–33.5)	14.0 (4.5–15.5)	16.0 (10.5–17.5)
Married	23.5 (29.0–16.0)	8.0 (4.0–12.75)	15.0 (12.0–18.0)
Unwedded	24.50 (17.5–32.0)	9.5 (5.0–14.75)	15.0 (12.0–18.0)
	*p* = 0.517	*p* = 0.245	*p* = 0.951
Coded psychosocial diagnoses:			
Family	Yes 20.5 (12.0–31.25) No 24.0 (19.0–31.0) *p* = 0.083	Yes 7.0 (2.0–12.0) No 9.0 (5.25–14.0) *p* = 0.113	Yes 13.0 (10.0–17.75) No 9.0 (5.25–14.0) *p* = 0.097
Work	Yes 23.0 (17.0–30.0) No 24.5 (17.0–32.0) *p* = 0.362	Yes 8.0 (4.0–13.0) No 8.5 (5.0–14.0) *p* = 0.512	Yes 15.0 (12.0–17.75) No 16.0 (12.0–18.75) *p* = 0.280
Biography	Yes 29.0 (21.0–35.5) No 23.0 (16.0–29.0) *p* = 0.002	Yes 11.0 (8.0–15.0) No 8.0 (4.0–13.0) *p* = 0.003	Yes 16.0 (12.5–20.0) No 15.0 (11.0–18.0) *p* = 0.020
Finance	Yes 24.0 (17.0–32.5) No 24.0 (17.0 – 31.0) *p* = 0.644	Yes 9.0 (4.0–15.25) No 8.0 (4.0–13.0) *p* = 0.366	Yes 14.5 (11.75–18.5) No 15.0 (12.0–18.0) *p* = 0.695
Any diagnose	Yes 24.0 (16.5–31.0) No 24.0 (17.0–31.0) *p* = 0.920	Yes 9 .0 (4.0–13.5) No 7.0 (5.0–14.0) *p* = 0.952	Yes 15.0 (12.0–18.0) No 16.0 (12.0–18.0) *p* = 0.623

## Discussion

The present study showed that perceived injustice levels closely correlated to depression, anxiety and stress. Moreover, there was a weak inverse correlation with quality of life. Conjunctions could also be demonstrated in the analysis of the coded diagnoses. The analyses of social factors revealed higher injustice scores in patients with ongoing pension application or current work leave and in patients with a lower educational level. Interestingly, among the coded psychosocial diagnoses, differences in the perceived injustice scores were shown only regarding biographical factors contributing to psychological distress. In the present study, IEQ values with median total IEQ were comparable to those in a previous study [5].

**Fig.1:**
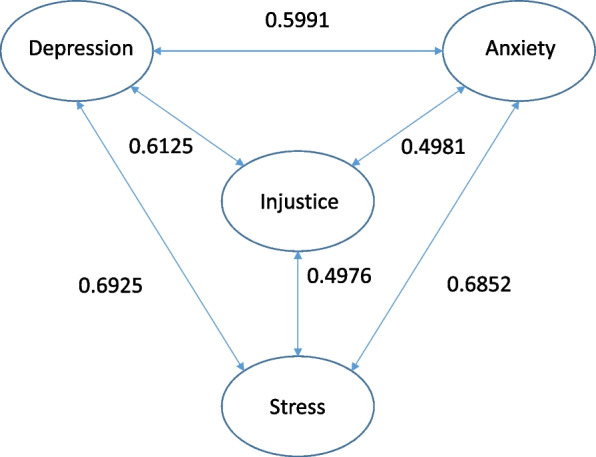
Correlations between anxiety, depression and perceived injustice

In fact, there is a close correlation between anxiety, depression, catastrophizing, stress and perceived injustice, as a number of studies have found [19]. A recent metaanalysis shows a correlation of the IEQ to a number of psychological variables such as pain catastrophizing, posttraumatic stress, anxiety, depressive symptoms, kinesiophobia and disability [12]. This correlation to anxiety, depression, stress and perceived injustice was confirmed also in the present study (Fig. 1).

When acute pain gradually turns into chronic pain, patients often experience a long cascade of frustrations due to ineffective treatments. The usual experience with acute pain is that of pain being rapidly relieved with or without therapeutic intervention. With this expectation, it seems logical, that the advent of chronic pain can lead to feelings of blame towards the treating physicians. This feeling might be reflected in the subscale blame/unfairness of the IEQ. Beyond that, there is an inverse correlation of the IEQ with pain acceptance. Martel et al. [23] found an inverse correlation of the IEQ and both subscales to Chronic Pain Acceptance Questionnaire scores. On the other hand, it is known that biographical stressors such as childhood trauma can also have a strong impact on chronic pain [35–37]. Therefore, it is also conceivable that a feeling of injustice has already prevailed in a patient prior to the onset of chronic pain and that this contributes to the perpetuation of pain, thus fostering pain chronicity. The present study adds some aspects pertaining to this question. Interestingly, we found that among the coded diagnoses differences in the IEQ scores were found only in patients with and without biographical factors. These results fit well with those of Zaidni and colleagues (2021). In a study of chronic pain patients, they used a meditation analysis to find that PI mediates the relationship between perceived childhood neglect and function [38].

Most of the social factors possibly contributing to chronic pain have not been correlated to the IEQ in previous studies. While some studies give information on social variables such as education, sick leave and family status to describe the sample characteristics [19, 22] only rarely correlations to the IEQ have been studied. Margiotta et al. found no significant correlation to occupational status and family status [20]. La Cour et al. examined correlations of the family status and occupational status and found that only missing current employment correlated significantly to the IEQ total and the IEQ blame but not to the IQ severity scale [21]. Correlations between the IEQ and current work leave or pension application have not been studied previously. In the present study, differences in the IEQ scores were found also in patients with and without work leave and in patients with and without pension application. Interestingly, we also found, that patients with higher educational level had lower values in the IEQ total and the IEQ severity subscale but not in the IEQ blame subscale. This stands in contrast to the finding that no differences were found among patients with and without contributing social factors regarding the work environment. Our result gives some hint that, on average, this might not contribute to the feeling of injustice to a considerable degree.

In an experimental study Sullivan et al. [7] examined the association of perceived in justice with pain behavior in patients with whiplash injury. Pain behavior can be subdivided into protective and communicative pain behavior. While protective pain behavior may aim at avoiding further organ damage or facilitate further remission after trauma, i.e. by avoiding load on the affected area, communicative pain behavior i.e. facial expressions such as grimacing or wincing or verbal pain expressions aim at communicating a persons` suffering to others. Sullivan et al. showed that the IEQ correlated only to protective but not to communicative pain behavior.

This notion is maybe also reflected in our results, as the IEQ correlated to depression, anxiety and stress but not to somatization disorder. Thus, those patients supposedly having a higher degree of communicative pain behavior may not have had higher IEQ levels, while those patients with a high degree of pain load, supposedly having a higher protective pain behavior also had higher IEQ scores. However, this interpretation must be seen with caution due to the low number of patients with somatization disorder.

In our study the IEQ total score was perfectly in line with the values given by Sullivan (2008), with 25% of the patient scoring above the 75% quartile from this reference. Yet, only 20% of the patients scored above the respective cut off for blame while 42.1% scored above the cut off for severity. Most of the patients in the present study were no trauma patients, who, in some cases, might have even had a reason to blame somebody for their destiny and who therefore might have higher values for the subscale blame.

The total values of the IEQ were perfectly in the range of the published values [5], which are derived from samples with musculoskeletal pain. We would have expected slightly higher values as our study was performed in a tertiary pain center with patients suffering from a high level of distress and usually having a long-standing pain anamnesis.

Some limitations have to be mentioned. First, the study was a retrospective analysis. This limitation applies in particular to psychosocial variables that relate to the past, e.g. biographical factors as a self-report bias could occur here. Moreover, the study does not analyze how perceived injustice may influence therapeutic interventions, such as multidisciplinary pain therapy for instance. This aim was beyond the scope of the present study but we are planning to address this in future. Further, the duration of pain was not included in the analysis as it was not part of the questionnaire. As many patients had a very long history of pain, the onset of pain could not be determined exactly in many cases. This impeded a closer analysis of this item. Moreover, as multiple items were studied a sample-size calculation was difficult to perform. Therefore there is a possibility of false negative results at least regarding single items. A detailed analysis of the individual underlying clinical pictures would have been gone beyond the scope of this study, and the large number of different diagnoses would have made a meaningful analysis difficult. In addition, some patients had two or more pain diagnoses from different localizations and different etiologies at the same time. We therefore attempted to classify the clinical pictures at least according to their primary localization.

Among the strengths of the study are the relatively high number of patients, a high return rate and the broad analysis of a variety of somatic, psychological and social factors possibly interacting with perceived injustice.

Besides confirming the close link between perceived injustice and depression and anxiety, the present study contributes information on how the feeling of perceive injustice interacts particularly with social factors. The concept of perceived injustice in chronic pain patients should be examined in further studies, as it might open a door towards further distinct (behavioural) psychotherapeutic interventions. To this end future research should strive to integrate contributing medical, biochemical, genetic, psychological and social factors.

## Data Availability

Data will be made available on reasonable request.
